# Unlocking the Paradox of Intercultural Collaboration in Integrated Community Care: An Interpersonal Dialogue

**DOI:** 10.5334/ijic.8596

**Published:** 2025-07-21

**Authors:** Mina Ishimaru, Naonori Kodate, Sanae Murai, Ghislaine Rouly, Antoine Boivin

**Affiliations:** 1 Department of Innovative Nursing for Life Course, Graduate School of Nursing, Chiba University, Japan; 2 Social Policy and Social Robotics, School of Social Policy, Social Work and Social Justice, University College Dublin, Ireland; 3 Public Policy Research Center, Hokkaido University, Japan; 4 Centre for Frontier Medical Engineering, Chiba University, Japan; 5 Institute for Future Initiatives, University of Tokyo, Japan; 6 Higashi-Chiba Wa-Wa-Wa no Kai, Chiba, Japan; 7 Canada Research Chair in Partnership with Patients and Communities, Centre de recherche du centre hospitalier de l’Universitéde Montréal (CRCHUM), Canada; 8 Canada Research Chair in Partnership with Patients and Communities, Centre de recherche du centre hospitalier de l’Universitéde Montréal (CRCHUM), Faculté de médecine de l’Université de Montréal, Canada

**Keywords:** interpersonal dialogue, cross-border learning, integrated community care, policy transfer, intercultural collaboration, relationships, socialisation

## Abstract

**Introduction:**

Intercultural collaboration in integrated community care faces a paradox. Some view community care as a ‘local craft’, intimately embedded within its socio-cultural context, and therefore it cannot be exported. Others view it as ‘interventions’ that are transferable and scalable, like other health innovations. This article proposes a middle-ground approach, highlighting the role of interpersonal relationships as a foundation for intercultural collaboration in integrated community care.

**Description:**

Over a five-year period, we pursued an intercultural collaboration between two integrated community care initiatives in Canada and Japan. Both initiatives are grounded in the principles of community empowerment, linkages across health and social care, and complementarity of lived experience and professional knowledge. Our collaboration evolved in three interrelated phases: 1) intercultural discovery and exploration; 2) intercultural relationship-building and strengthening; and 3) intercultural explicitation and influence.

**Discussion:**

While the implementation science literature largely focuses on cognitive processes of knowledge exchange, our experience highlights deeper relational dimensions that are essential to intercultural learning and impact across community care initiatives, including socialisation among collaborators, beyond their professional roles and identities.

**Conclusion:**

Relational and socialisation processes should be recognised, nurtured and valued as integral components of intercultural collaborative efforts in integrated community care. Knowledge gained from this experience can inform cross-cultural efforts to support the global integrated community care movement.

## Introduction

### Background

While health systems worldwide are struggling to move away from disease-oriented, biomedical models of care, integrated care is growing into an international movement [1,2]. Interest in collaboration is exemplified by the growth of international organisations and journals dedicated to integrated care [3,4,5,6,7].

Within this broad movement, integrated *community* care seeks to empower people and communities in improving their health and well-being. Integrated community care is relationship-oriented, asset-based and community-focused. It seeks to support people and communities in achieving their own goals. Integrated community care moves beyond professional healthcare services, to act on social determinants of health such as social inclusion and empowerment. Finally, it is highly participatory in nature, recognising communities’ knowledge and strengths, and working with them as partners in health.

Intercultural collaboration in integrated community care faces a peculiar paradox. Some argue that community care is a ‘local craft’, intimately embedded within its socio-cultural context, and cannot therefore be ‘exported’ elsewhere. Others view it as ‘interventions’ that can be transferred, scaled-up, and adapted to other cultures and countries. This article proposes a middle-ground approach – foregrounding the role of interpersonal relationships in intercultural collaboration – based on a five-year project across two integrated community care initiatives in Japan and Canada.

### Problem Statement

#### Challenges of Scaling Innovations across Contexts

In the implementation science literature, various challenges of disseminating innovations across contexts have been raised [8,9,10]. May et al. [9] emphasises the significance of ongoing interactions among participants and contexts. In the ‘real world’, translational efforts in healthcare interventions take place in open, complex, and dynamic systems. Therefore, transfer of knowledge from one local context to another is non-linear, emergent, and dynamic [11].

In public policy, policy transfer refers to the process by which actors borrow policies created in one setting to develop programmes and policies within another [12,13]. Policy transfer may involve the adaptation of policy tools used in one country or culture to another. Some call this process ‘lesson drawing’ and ‘policy diffusion’ by policy brokers and policy entrepreneurs.

Greenhalgh and Paoutsi argue that what works smoothly in “setting A” will operate awkwardly (or not at all) in “setting B”’ [8]. In contrast to the conventional mechanical approach of exporting and adapting knowledge, implementation must be seen as adaptive change to different socio-cultural settings.

Some scholars are altogether critical of the possibility of scaling social innovations across contexts. Godrie argues that innovations are often considered in terms of their technological, patentable and marketable aspects, rather than its human and social aspects. This is based on the premise that local innovations need to be scaled up – when their success often rely precisely on the fact that they are rooted in local context – making their generalisation problematic [14]. Echoing this position, the authors of *Scaling Impact Innovation for the Public Good* propose that, instead of exporting interventions from one context to the other, our focus should be shifted towards scaling social impact [15]. In other words, we must pay greater attention to clarifying underlying goals and supporting inclusive coordination at optimal scale. In contrast to these arguments, the concept of Scaling Deep supports the idea that durable change can be achieved primarily by transforming people’s “hearts and minds”: their values, cultural practices and relationships [16].

These paradoxes and tensions around implementation and scaling are particularly acute for integrated community care. Integrated community care is a deeply local and human endeavour: It builds on small-scale initiatives rooted in geographically-defined local communities. At its heart, integrated community care is also about connecting people: patients, community members, healthcare providers, and local policymakers. Given complexity in care delivery in communities, the key lies in looking past the simple elements of a system to embrace complex system functions and processes [17,18]. Improvement therefore involves tailoring to context and harnessing local agents’ self-organising and sense-making capacities [19].

#### The role of culture in integrated community care

Cultural differences are particularly relevant to consider in the scaling of “human-powered” and “relationship-focused” innovations such as integrated community care. Hofstede defines culture as ‘the collective programming of the mind that distinguishes the members of one group or category of people from another’ [20, p. 9]. Culture includes deeply embedded values, beliefs and practices that are shared by a group. Culture can operate at the level of a community, a profession, an organisation or a nation. Within the context of integrated community care, these cultural norms, beliefs and practices can include who is considered a caregiver in the community, who acts as legitimate leaders, and whether health and social care activities are individually or collectively oriented. Hofstede views culture as largely implicit and stable, although it can be adapted over time. This means that implicit cultural norms can potentially be made more explicit and reshaped over time.

Studies about international scaling of integrated care tend to view international collaboration as a cognitive process of knowledge transfer, adaptation and use. It also tends to be underpinned by cultural assumptions about the distinctive roles of ‘innovators’, ‘researchers’, ‘implementers’, and ‘end users’ [21]. For example, a recent international scoping review of integrated community care initiatives found that the majority of projects tend to position researchers in the ‘evidence-based designer role’, professionals and policymakers in the ‘implementer and norm-setting roles’ and community members in a ‘knowledge users’ role. Oftentimes, these key actors are not part of the same ‘team’, and their functions tend to be tightly separated. Therefore, they do not work as ‘us’, and when they do, they tend to hold a very strict sense of their assigned roles, identities, and division of labour.

Other authors have questioned this strict division between who creates and uses knowledge in innovation processes. Nonaka and Takeuchi proposed the SECI model of knowledge creation (Socialisation, Externalisation, Combination, and Internalisation) in which collaborative work on innovation facilitates the explicitation, exchange and integration of knowledge, including cultural norms and values [22]. The key mechanism is the processes of socialisation (interpersonal relationship-building and sharing of experiences and worldviews), externalisation (articulation of tacit knowledge into explicit forms), combination (integration of synthesis of knowledge from different sources) and integration (internalisation and application of new knowledge in practice). The study on intercultural collaboration between France and China by Lievre and Tan highlights the importance of socialisation in knowledge exchange activities across distinct cultures [23].

In this article, we reflect on the process and impacts of intercultural collaboration in integrated community care. The article builds on a five-year (2020–25) collaboration between two integrated community care initiatives embedded in culturally-distinct settings: Caring Community (Canada) and Wawawa-no-Kai (Japan).

The article is structured in three main sections.

1. First, we describe the intercultural collaboration practices between the Canadian Caring Community and the Japanese Wawawa-no-Kai initiatives.
2. We then describe the process and outcomes of intercultural collaboration through first-person lived experience accounts of key actors engaged in the collaboration.
3. Finally, we discuss key learnings and principles that can inform future research and practice on intercultural collaboration in integrated community care.

In line with participatory research traditions, this project brought together academic researchers, health professionals, patient partners, and citizens working together in a shared process of scientific collaboration. This participatory research approach emphasises the importance of relationships among people drawing from different sources of expertise to create, share, and apply new knowledge. This approach is also coherent with the SECI model of intercultural knowledge creation through ongoing processes of socialisation, explicitation, combination and integration. It also reflects the principle of gemba (‘actual site/field’ in Japanese), which highly values expertise, experience, and skills of those working ‘at the coalface’ [24], including people with lived experience of integrated community care. In line with these approaches, the article blends different voices: third-person reporting of the literature with first-person accounts of the authors’ experiences of intercultural collaboration.

### Ethics Approvals

This project was embedded in the Caring Community research program, approved by the research ethics board of the Centre intégré de santé et de services sociaux du Centre-Sud-de-l’Île-de-Montréal (#2020–564, DIS-1819–77), as well as the ‘Adapting the Caring Community Concept to Japanese Practice: A Comparison of Case Studies of Activities in Canada and Japan’ approved by the research ethics board of the Graduate School of Nursing at Chiba University, Japan (NR4–77).

### Intercultural Collaboration Description

In 2020, an international collaboration was launched between Japan and Canada to explore cross-cultural adaptation of integrated community care. We studied collaboration across 2 “cases” of integrated community care implemented in different cultural contexts: Caring Community (Canada) and Wawawa-no-kai (Japan). Both initiatives are grounded in the principles of integrated community care.

Initiated by a Japanese academic researcher (MI), the collaboration engaged with the patient and physician co-founders of the Caring Community initiative (GR and AB) and a Japanese citizen (SM) who co-founded the Japanese Wawawa-no-Kai initiative. Cross-cultural research and collaborations across the Canadian and Japanese teams was facilitated by a Japanese-born social scientist from University College Dublin in Ireland (NK), who acted as a language and cultural translator and facilitator.

According to Hofstede’s taxonomy of national culture, Canada and Japan have various cultural differences (eg. more individualistic or collective orientation toward community care) [25]. While conscious of the potential danger of sweeping generalisations, we acknowledged some of these cultural differences were reflected in practice. For example, Wawawa-no-Kai fosters community engagement primarily through group-setting activities, while Caring Community emphasises one-to-one support by community members acting as peers.

#### Caring Community (Canada)

Caring Community is a participatory research program on the integration of peers in community care teams [26]. Caring Community is grounded in a partnership model of integrated community care. It supports integrated community care through alliances between people with lived experience and healthcare professionals. Caring Community positions peers – people with significant lived experience of health and social challenges – as accompaniers and ‘bridgers’ between people, healthcare teams, and communities.

Caring Community was co-founded in 2016 by a patient partner (GR) and a clinician-scientist (AB), in one of the most socially deprived neighbourhoods of Montreal, Canada. A first peer with lived experience of multiple chronic conditions was integrated as part of the healthcare team of a community-based primary care clinic. This peer accompanied people of different ages (25 to 90 years old), gender, ethnic background, health conditions and social challenges (mental health, chronic pain, social isolation, poverty, housing insecurity, cancer, end-of-life). In 2020, a second peer with lived experience of homelessness was integrated in another community health clinic to support outreach and accompaniment of people experiencing homelessness: in the streets, shelters and within the healthcare system. In 2022, a third healthcare team integrating indigenous peer-navigators accompanying Indigenous people facing complex medical and social issues was included in the participatory research program. In 2024, three other healthcare teams working with peers with lived experience of mental health, substance use and with migrants joined the program.

#### Wawawa-no-kai (Japan)

Wawawa-no-Kai is a voluntary group set up in 2015 by residents in Higashi-Chiba District (Chiba, Japan), a suburban area of Tokyo, and founded through a partnership between community leaders (SM and Katsunori Murai), nursing academics (Prof. Mariko Otsuka (President, Nagano College of Nursing) & MI), municipality, and citizens. Facing the limit of self-help, family care, and neighbourhood associations, several residents voluntarily sought to create a support network and began to organise themselves. With a super-aged society, integrated community care began to be promoted by public authorities. Deep-seated psychological and socio-cultural barriers had existed and denied the super-aged access to care. From the perspective of care provision, interprofessional collaboration was hard to establish in communities. Reaching out to a local municipality and academics, Wawawa-no-kai had one primary goal of “enabling ‘living the life we/older adults wish to live’ in our own town” [27].

Once the group was established, community members identified ‘common problems’. Subsequently, regular workshops were organised to raise awareness and create psychologically safe space for mutual help. The community-development approach adopted by the group includes a community salon for socialising and freely discussing their citizens’ concerns. Local doctors and welfare officers are also invited to give sessions. Although the majority of members were (retired) men at the outset, they adopted a ‘shared leadership’ posture [28] and stayed active in a serving role (e.g. offering coffee to guests) as well as leading functions. Another prominent example is the ‘greeting’ (aisatsu) project, which aimed to build a positive and friendly (intergenerational) relationship among local residents. Every morning, senior community members stand in front of their houses, wishing passers-by good morning. This activity culminated in the naming of a street (Higashi-Chiba Aisatsu Road), funded by the municipality under the banner of Chiba City’s 100th anniversary since its foundation.

#### Intercultural Collaboration Processes

**Figure 1:**
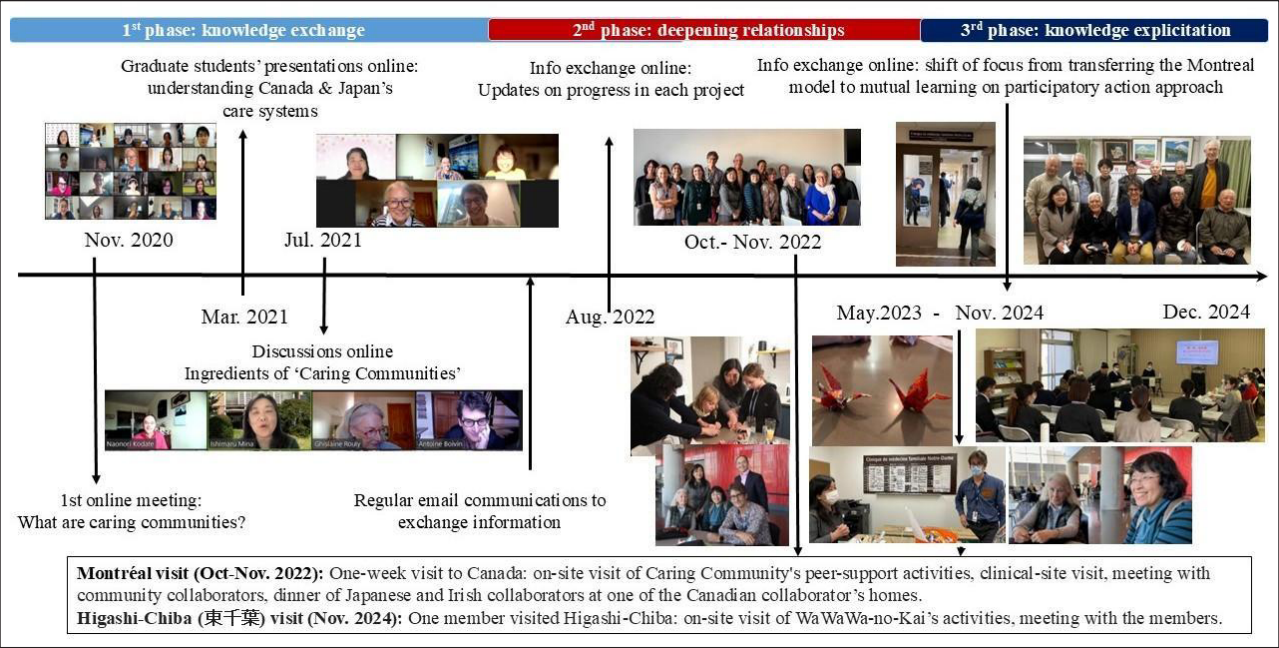
Timeline of the intercultural process (2020–2025).

The intercultural collaboration between Caring Community and Wawawa-no-kai evolved over a period of five years (2020–2025), over three main phases: 1) knowledge exchange; 2) deepening personal relationships; and 3) knowledge explicitation (Figure 1). In the first phase (2020–2022), intercultural collaborators held a series of online meetings and seminars to learn about each other’s initiatives, differences between the Canadian and Japan healthcare systems, and community engagement practices. The second phase (2022–2024) was primarily conducted through 2 onsite visits to Canada (november 2022) and Japan (november 2024), which included on-site participation in integrated community care activities, meeting with community collaborators, and social activities. The third phase (2023–2025) included a series of follow-up meetings between collaborators to reflect on similarities and differences between both initiatives, articulate what had been learned, and communicate it explicitely.

The SECI processes of socialisation (e.g. relationship-building), explicitation (e.g. expliciting and exchanging information about each community care initiative), combination (e.g. sharing observation and synthesis of learning across teams) and internalisation (e.g. integration of new insights and approaches in local community care practices) were iteratively embedded into these different phases.

#### Knowledge explicitation methodology

Knowledge explicitation aimed at transforming tacit knowledge generated throughout the process of intercultural collaboration into explicit knowledge that could be shared collectively, applied locally, and communicated more broadly to others. In accordance with the participatory research approach, there were overlaps between who were the actors of intercultural collaboration and who studied the intercultural collaboration processes. All of those involved as authors in this article were both actors and observers.

The knowledge explicitation process evolved in three stages: 1) personal observations and reflections; 2) sharing among local national team members; 3) cross-cultural discussions among Japanese, Canadian and Irish collaborators to contrast and discuss different viewpoints. Throughout the intercultural collaboration process, each of the authors took personal notes of their observations, feelings and learnings about themselves, community care initiatives, and the intercultural collaboration process. In total, we held 11 intercultural research team meetings in English to share our observations and learning. After those meetings, we shared written notes about common and contrast observations. Approximately 20 complementary national meetings in Japanese (Japan and Irish team) and French (Canadian team) were held among national teams to ensure understanding and discuss local implications. From 2023 to 2025, four team members from the Canadian and Japan teams were invited to put in writing their own personal story of lessons learned regarding the process of intercultural collaboration in integrated community care. These drafts were shared and discussed in team meetings to identify common and contrasting themes. The Irish author took an “outsider’s perspective” to facilitate the process of knowledge explicitation and intercultural understanding. He drafted the introduction and discussion section to put the observations in dialogue with the international published literature.

The following result section presents the observations from four of the authors directly engaged in the intercultural collaboration, followed by a discussion about how these learnings relate to the wider published literature on intercultural collaboration in integrated community care.

## Experiencing the Intercultural Collaboration Process

### Ghislaine Rouly: Caring for Oneself, Caring for Others, Caring with Each Other

Ghislaine Rouly is a 78 year-old patient. She co-founded the Caring Community in Canada, where she works as a peer mentor: a person with lived experience, accompanying other people in their life journey. Her dream of becoming a doctor was cut short by diseases early on in her life. Yet she never stopped caring for others, differently. Without official title nor diploma, Rouly (re)invented over decades her own way of caring, listening with the heart, sharing useful experiences when needed, and modeling hope that one can live a full life despite severe illnesses.

After many years working alone, she started building alliances with healthcare professionals:

*“In 2007, my third cancer left me with a need to do more. I started teaching medical students. In 2015, while giving a lecture, I had the chance to meet Dr. Antoine Boivin. Interested by my life trajectory, he approached me. I met an incredible human being. A doctor that only in dreams you have. A person who dares to do what he is teaching. A researcher who goes where few dares to go. A man who thrives on making the invisible visible—mostly the most vulnerable of all patients. Together we ‘care’ for patients. Patiently, at the speed of trust, we built a program where patients like me are recognized as caregivers. Each with our own knowledge, his from medical sciences, and mine rooted in experience. We are complementary in our approach to care.”*

From this meeting between a “caring patient” and a “caring doctor” was born the Caring Community initiative, where peers are integrated in community healthcare teams. Starting from a pilot initiative, Caring Community slowly evolved into a larger community-based research program.

*“Since 2016, we have expanded, adding psychologists, social workers, trainees, nurses, other doctors, etc. Together we work. I became a ‘colleague’; one of them. My knowledge is recognized, sought out, and greatly valued and appreciated not only by the team but mostly by the patients. As the years go by, we write articles and hold conferences not only in Canada but internationally.”*

While the Caring Community initiative initially faced resistance locally, it generated surprising interest from a few international colleagues engaged in similar initiatives. Rouly and Boivin were invited to publish a short paper on their experience in the British Medical Journal [26], which caught the attention of a Japanese team who contacted them:

*“In 2020 we were approached by a Japanese team. To do what exactly? Our cultures seemed so different; how could we work together? I was excited and delighted by their curiosity and felt strong in the knowledge that if we managed to truly communicate through our values, it would be a magnificent adventure—a human adventure. Because what we do, Antoine and I, is just that. Human relationships. Caring at the most humanistic level: from the heart, with humility, devotion, and sincerity. Not only do we work at the speed of trust but also at the speed of the patient”*.

From this initial feeling of distance at the “knowledge exchange” stage of webinars, grew an increasing curiosity to learn from one another during the “relationship-building” stage of the collaboration and the on-site visit in Montreal. Rouly was particularly struck by her meeting with a citizen-partner from Japan (Sanae Murai) who co-founded the Wawawa no-kai initiative:

*“Our Japanese colleagues decided to come to see us. What a great privilege to finally, after two years, meet all of them in person; Mina-sensei, Nao-sensei, Mrs. Murai. It was like love at first sight. We spoke the same language, one of the hearts. Mrs. Murai and I are doing the same work, differently, in the sense that she works with a group, and I work with one person at a time. But our approaches are the same. We exchanged and transferred back and forth our knowledge, sharing stories, laughing, and being humbled by each other’s work.”*

For Rouly, the pinnacle of this on-site visit was a dinner in Boivin’s house. For her, this was a pivotal moment where “international collaborators” became “team members” and “friends”. After the Montreal visit, something shifted in the way international collaborators related to one another. Rouly highlighted a deeper sense of cooperation:

*“Now each team is back in its own country; the masks are back on, but they are different somehow. We know who’s behind it now. The work is more fluid, the exchanges feel different, we are more daring in our approach, the openness is wider, mixed with humor shared only among friends. Because this is what the teams have become—friends. This is a unique situation in research where so much competition exists. And somehow we do compete at a different level than scientific. We compete to alleviate the suffering. Mrs. Murai and I compete to better develop techniques in listening, in interpreting non-verbal language, in discovering the various needs of the people we care so much about. Both Mrs. Murai and I compete to better learn and exchange later on. It is a competition of the mind and heart with each other, in order to give the best in a group or at a more personal level.”*

Finally, Rouly reflects about what can and cannot be transferred and scaled across cultures based on this international experience:

*“Caring Community, as we call our program, does not have a recipe to repeat itself. Because what we proposed is something that has existed since the beginning of time. We are just repeating what has been done naturally everywhere, anywhere you find vulnerability and suffering. Mrs. Murai deals with it in Japan. Myself, thousands of miles away. But together we are giving the ultimate gift: time*.

*Five years later, it is interesting to realize the influence that we still have on each other. Mrs Murai works with a group of people, I, one-on-one. But during the Fall of 2024 I organized a picnic, inviting all of the patients I care for, to join me in a beautiful park. I was expecting at most 2 or 3 persons: the majority of them joined me. They didn’t know each other. Some walk with difficulty, others with canes, some have severe mental issues, others chronic pains. Some are in their 20s, others in their 80s. But they all came*.

*It was a labor of love, respect, curiosity done by each and every one of them. For some it was the first time in a very long time that they came out of their home, for others just having to speak with strangers was in itself a huge challenge. I witnessed thousands of little miracles that day. My group is a little sample of what the planet looks like in terms of diversity, gender, religion, ethnicity, size, language, culture and somehow we were, for one day, able to put all our differences aside to concentrate on our similarities.”*

### Antoine Boivin: Caring with Communities across Cultures

Antoine Boivin is a community physician in Canada. Early on in his career, he adopted a “participatory” practice:

*“My whole life is about bringing people and ideas together: through participatory music, medicine, and research. For me, meeting a new person is like travelling to a new country.”*

Meeting Rouly flipped his perspective about patients as *caregivers*:

*“My relationship with Ghislaine transformed the way I practise medicine. In school, I was taught that patients are people we care ‘for’. Ghislaine however was a patient caring ‘with’ others. She accompanies them as a peer, based on her own lived experience.”*

Caring Community started in a primary care clinic where Rouly and Boivin started caring for people together. It quickly took an integrated community care perspective when they started connecting with other community members:

*“After experimenting in our own clinic, we connect with other teams working with people with lived experience of homelessness, substance use, mental health, migration and Indigenous health. We started to support and learn from one another.”*

Seeing the Caring Community as an organic, locally grown initiative, Boivin reports an initial discomfort at the idea of “exporting” Caring Community to Japan:

*“When Mina-sensei and Nao-sensei wrote to us in 2020, they told us that they wanted to implement a Caring Community in Japan. I felt intimidated. First, because I did not know anything about Japan. Second, I felt that the Caring Community was more a set of values than a model to be exported. How can we export relationships of care?”*

Boivin describes the initial phase of knowledge exchange as a “taming process”, allowing each person to slowly learn about one another:

*“We did what we do best: engage in dialogue with our new Japanese colleagues. We exchanged about our healthcare systems, communities and approaches. But most importantly, we slowly learned to trust each other. What originally felt like a transactional interaction slowly shifted to a relationship driven by curiosity (Who are you? What is community care for you? What can we learn from our differences?).”*

Boivin points at the importance of “cultural translators” to facilitating mutual understanding and trust among the Canadian and Japan teams:

*“Nao and Ghislaine both acted as ‘bridgers’, ‘diplomats’, and ‘translators’. Nao was born in Japan and lives in Ireland: his presence was key to understanding differences in health systems and cultures. Ghislaine lived in Japan for a year and also helped our team interpret cultural cues and non-verbal languages between us.”*

He also points at the pivotal role of informal face-to-face meetings, outside of usual work settings to transform relationships among intercultural collaborators:

*“A pivotal point in our relationship was a one-week visit by Mina, Nao, and Mrs. Murai in Canada. This was the moment when we all ‘removed our masks’ (as patient, physician, researcher, Japanese or Canadians) and simply became ‘people’. During dinner at my house, Mina and Mrs. Murai started doing origami with our kids. We shared life stories and recognised each other as fellow human beings, beyond our differences.”*

*Boivin points at the importance of understanding the broader cultural context in which each integrated community care initiative is rooted:*

*“Another key moment was my own visit to Japan to meet with Wawawa-no-kai community members. Public spaces are very formal in Japan: you rarely see people greeting strangers in the street. Yet, elderly people engaged in Wawawa-no-kai took the initiative of greeting young children every morning on their way to school, thus fostering intergenerational connections. The movement grew in such a way that the local municipality renamed a street ‘Assatsu Road’: the ‘greeting street’. This taught me that community engagement is not only rooted in culture, but can also contribute to changing it.”*

Boivin reflects on the connections that remained after these visits. He describes this as a process that moves beyond knowledge exchange, into a process of deeper mutual cooperation:

*“What we have built through personal relationships are invisible rhizomes nurturing local communities across borders. Rhizomes are underground root systems connecting two plants that can feed and support each other. It is how strawberries grow. It is the same invisible roots that connect our communities, beyond our differences.”*

He also points at how collaboration transformed his own cultural views about care:

*“A few years ago, I viewed myself as a physician caring for one person at a time. Now, I see myself as a community physician, facilitating community connection and empowerment. I no longer see individual care and community care as two opposites: they are two sides of the same coin.”*

### Mina Ishimaru: A Japanese Public Health Nurse-Researcher’s Perspective

Mina Ishimaru is a Japanese researcher and public health nurse with expertise in health promotion and community engagement for aging citizens. Engaged for a number of years with the Wawawa-no-kai project, she recognised common principles with the Caring Community project. She first reflects on the initial stage of collaboration :

*“Our early online meetings aimed at knowledge exchange via my graduate students’ presentations. We shared the baseline information, e.g. Japanese and Canadian healthcare systems, the caring communities, and the community-integrated care efforts in Japan.”*

Despite familiarity with *studying* community engagement, Ishimaru was new to engaging community members in her research team. A key early impact of the intercultural collaboration was the integration of an engaged citizen in the research team. *Also*, Ishimaru reflects on direct interaction with Caring Community leaders changed her understanding, revealing initially uncovered similarities:

*“I originally thought that Ghislaine-sensei and Antoine-sensei viewed the community as a group, not geographically defined. But learning about its adaptation to homelessness showed me that a community can be a group, a local, and have social capital (relationships, trust, and reciprocity). Afterwards, we found more similarities with Wawawa-no-kai.”*

Ishimaru believes the collaboration unveiled not just the “what” but the “why” of engagement, highlighting intention. This shifted inquiry:

*“In Canada, through the question ‘Who are you?’ showed me each person has unique experiences and thoughts, and their ‘original motivation’ is key. Talking and listening deepens bonds. between the people and the relationship can deepen through those activities. Discussing ‘why they started’ and ‘the future goals’ felt important.”*

A significant mutual influence was the individual versus group approach. Ishimaru recognised one-to-one support in the group-based *Wawawa-no-kai*:

*“In Japan, we say the individual moves toward the community and vice versa. Wawawa is group-based, but Ms. Murai emphasises individual identity, not just group function. A resident bringing his wife with dementia to the salon, where members warmly welcomed them, was memorable. Through group-based, Wawawa values individual relationships and connects those with age or disability-related engagement difficulties. We realised Ms. Murai’s and Ghislaine-san’s activities align…”*

*“After the trip to Canada, I and Ms.Murai began our community nursing practicum at the Higashi-Chiba community center. Aiming to encourage Wawawa-no-kai members and residents to live as they are in the community (i.e. truly integrated community care), we included individual storytelling alongside group activities. By Sharing stories, we sought to create a place for expressing intentions and understanding motivations, fostering engagement. We also wanted to convey to the students the importance of respectful individual care within group activities.”*

### Sanae Murai: a resident wondering ‘how best to live in our local community as who we are’

Sanae Murai is a senior citizen in the Japanese community of Higaschi-Chiba. In collaboration with her husband Katsunori, she co-led the Wawawa-no-kai initiative to bring together older people in her community to “live their best life, as who they are, in their community”. Murai has a community-orientation to her work, facilitating group discussions among community members to identify common problems and implement solutions together. From the outside, her work appeared quite different from one-to-one support offered by Rouly in Canada:

*“Among people who had many conversations with people in Canada, there were two words that shook my heart. One was from Ghislaine-san, a patient partner, who said, ‘Ms. Murai and I are very similar. We are doing the same work’. She invited me to her own peer counselling session and introduced the activities of the Wa no Kai, and after spending about an hour with the current patients, she gave me her impression. ‘The big difference is that I am dealing with one patient as one person, but you are working with a group of people (residents)’ and ‘The same thing is that we are working with respect to each person’s thoughts, with shared values’.”*

Murai was also struck by the shift in focus from “what do you do” to “who are you”:

*“The second question was asked by Antoine-sensei: ‘Who are you?’ As I responded to these questions, I asked myself, ‘Why am I doing what I do for Wawawa-no-kai now? What is it that is important to me?’ Through this visit, the meaning (value) of our activities slowly became clear to me. Friends of friends are friends. Starting with a greeting, the number of acquaintances grows. Through some chance, they find the same experience and what they want to do. And then they do something together. Through these activities, people recognise each other and care about each other. In this way, many ‘connections’ that recognise and care about each other are formed in this community. I think this is the ‘Japanese Wa-style Caring Community’, isn’t it?”*

Reflecting on the impacts of the relationship-building stage of the intercultural collaboration, Murai highlights its effect on her own sense of identity and empowerment:

*“When we gave a presentation in Montreal, I realised that we (Wawawa-no-kai) have been or could become what is called ‘patient…/citizen partners’. We have our own concerns about our own ageing: ‘How long will I be able to organise and participate in these activities?’ However, no matter what state we are in, our position as ‘the active party/self-help group’ in the ‘caring community’ will not change. There must be something we can do as citizen partners, no matter…the situation. I believe that this is where both the individual and Wawawa-no-kai as a group can find the value of their existence. This must be the essence of local communities supporting integrated care.”*

Finally, beyond individual self-realisation, Murai points at the realisation that alliances among citizen and other community partners can effect change:

*“At the same time, we realised that…to make the community a safer place for us, it is important to get to know more residents, to create a place where each person can demonstrate their strengths, and to request help from other organisations (including universities) and specialised units in municipalities or public organisations. I hope that we can make more important connections with professionals who are willing to be ‘supporters/companions’ to help… individual residents address their own needs, and move forward steadily and surely towards building a caring community for integrated care.”*

## Discussion

It remains rare to find concrete examples of learning from each other’s model and implementing those lessons collectively in different cultural settings. Learning from community care initiatives in Canada and Japan unearthed many common elements, which have been highlighted in previous research [23]. This article reflected on the process and impacts of intercultural collaboration in integrated community care, based on empirical observations from a five-year collaboration between Japan and Canada. Grounding the analysis on a culturally-adapted version of the SECI model helped identify key processes at play in intercultural collaboration. Taking a cultural lens on international collaboration offered a broader perspective on integrated community care, looking not only at how knowledge is being shared but also how similarities and differences in values, beliefs and practices are being made explicit, shared and internalised.

Within this project, culture manifested itself on at least two distinct levels [25]. First, prior to the collaboration, common principles of integrated community care were interpreted and practised differently between cultures (e.g. central focus of activities on achieving personal goals in one locality and community goals in the other). Second, the process of international collaboration helped articulate these tacit cultural assumptions into explicit differences that could be explored with curiosity. Third, we observed greater cultural understanding and selective influences as an outcome of intercultural collaboration (eg. exploration of individual and collective-based activities across both sites). Our findings point out the significance of the relational and socialisation components of intercultural collaboration. This very much aligns with Lievre and Tang’s findings [23].

### How Can We Move Beyond ‘Lesson Drawing’ and ‘Knowledge Transfer’?

While there is now abundance of evidence in the realm of integrated care to support social determinants of health (the conditions in the environments where people are born, live, learn, work, play, worship, and age), efforts are still ongoing and often local. This indicates that merely looking at how other people successfully introduce and manage a type of integrated care in different socio-cultural contexts has not brought about policy diffusion on the ground. How can we improve this?

Importantly, we must develop a deeper understanding of ‘agency’ (versus ‘structure’) [29]. Agency signifies the capacity of individuals with the power and resources to overcome challenges and fulfil their potential. As mentioned above, socially shared meanings that derive from the interaction of social beings (i.e. culture) are often embedded in institutions and constrained by ‘structure’ (e.g. a formal health care policy or delivery model). However, a set of actors with a strong sense of mission and motivations can embark on new initiatives, including international collaboration.

Therefore, if the human and interpersonal aspects of pioneering community-based integrated care projects are disregarded, the probabilities of effective learning from each other’s model or initiative beyond socio-cultural differences would be hampered. Here, it is also worth exploring similar and dissimilar roles that ‘shared leadership’ [28] played in the two different community settings in our future research.

### Intercultural Collaboration in Integrated Community Care as Complexity Science

As described above, ‘What we have built through dialogue are invisible rhizomes nurturing local integrated community care across borders’. The dialogue was between researchers and patient-citizen partners from Japan and Canada, who have lived in different cultures, healthcare systems, and social care systems, and who live far apart. Through these dialogues and deepening relationships, each party learned and made sense of actions and behaviours that enabled and promoted integrated community care. As reported here, the impacts of personal and professional relationships transcended cognitive learning, including mutual recognition and engagement toward each other.

Recent studies suggest that integrated community care, implemented locally, should be expanded to increase impact so that positive change can be created, deepened and sustained in society [15,16,17,18,19]. What we have learned through an interpersonal dialogue across the two cultures was not a shortcut to finding the most efficient method of realising integrated community care, but what is real and authentic on the personal level, because we learned not only what we have been doing and why, but also who we are and how we relate to each other.

Motivations and values, as driving factors for the activities, were originally implicit (‘hidden’), but through the interpersonal dialogues, they were made explicit. The learning among the five participants generated new ideas, and sparked energy in their mutual activities in each location. Although we did not consciously use complexity science [13] to instigate our activities or analyse ourselves, abundance of evidence in our article indicates that in international collaboration, dialogue is the trigger, knowledge is created and shared in the context of the relationship, and that sharing that knowledge in each context could advance the change process in integrated community care.

#### Lessons Learned

- A set of actors with a strong sense of mission and motivations have the potential of embarking on intercultural collaboration in integrating community care.
- Interpersonal dialogue, socialisation and relationship-building are key conditions for constructive and situated learning on integrated community care, across cultures.
- The value placed on the human and interpersonal aspects of pioneering community-based integrated care projects could be an effective catalyst for enacting one’s learning within each other’s model and strengthening initiatives across socio-cultural differences.
- The dialogues, through which each party made sense of actions and meanings, enabled and promoted integrated community care.
- The interpersonal dialogues were made explicit, and this is where a shared ‘meaning’ emerged, reflected in subsequent developments of respective projects.

## Conclusion

When we face the paradox of cross-national learning and collaboration, an interpersonal dialogue approach can be a very effective way of reinvigorating integrated care community projects in a local context. The results from our project suggest that dialogue in intercultural collaboration can be a catalyst for the creation and sharing of knowledge in these relationships. What’s more, they indicate that the sharing of this knowledge in each context can internalise this knowledge and advance the transformation process of integrated community care in the localities concerned.
